# Empirical Study on Designing of Gaze Tracking Camera Based on the Information of User’s Head Movement

**DOI:** 10.3390/s16091396

**Published:** 2016-08-31

**Authors:** Weiyuan Pan, Dongwook Jung, Hyo Sik Yoon, Dong Eun Lee, Rizwan Ali Naqvi, Kwan Woo Lee, Kang Ryoung Park

**Affiliations:** Division of Electronics and Electrical Engineering, Dongguk University, 30 Pildong-ro 1-gil, Jung-gu, Seoul 100-715, Korea; westpan@dongguk.edu (W.P.); jung4759@gmail.com (D.J.); steve10@hanmail.net (H.S.Y.); exexzz@naver.com (D.E.L.); rizwanali@dongguk.edu (R.A.N.); whrrk2001@naver.com (K.W.L.)

**Keywords:** gaze tracking, optimal viewing angle and DOF of camera lens, empirical study, accuracy, user convenience and interest

## Abstract

Gaze tracking is the technology that identifies a region in space that a user is looking at. Most previous non-wearable gaze tracking systems use a near-infrared (NIR) light camera with an NIR illuminator. Based on the kind of camera lens used, the viewing angle and depth-of-field (DOF) of a gaze tracking camera can be different, which affects the performance of the gaze tracking system. Nevertheless, to our best knowledge, most previous researches implemented gaze tracking cameras without ground truth information for determining the optimal viewing angle and DOF of the camera lens. Eye-tracker manufacturers might also use ground truth information, but they do not provide this in public. Therefore, researchers and developers of gaze tracking systems cannot refer to such information for implementing gaze tracking system. We address this problem providing an empirical study in which we design an optimal gaze tracking camera based on experimental measurements of the amount and velocity of user’s head movements. Based on our results and analyses, researchers and developers might be able to more easily implement an optimal gaze tracking system. Experimental results show that our gaze tracking system shows high performance in terms of accuracy, user convenience and interest.

## 1. Introduction

Gaze tracking has recently become a very active research field that has applications in many different fields, including human computer interfaces (HCI), virtual reality (VR), driver monitoring, eye disease diagnosis, and intelligent machine interfaces. In detail, various applications using gaze tracking system are shown such as user–computer dialogs in desktop environment [1], head-mounted gaze tracking for the study of visual behavior in unconstrained real world [2,3,4,5], and for the study of oculomotor characteristics and abnormalities in addition to the input devices for HCI [6], eye-typing system for the user with severe motor impairments [7], and HCI in desktop environment [8,9,10,11,12,13,14,15,16,17,18,19,20]. Gaze tracking systems can find the position a user is looking at by using image processing and computer vision technologies. Various kinds of gaze tracking devices, wearable and non-wearable and with single or multiple cameras [1,2,3,4,5,6,7,8,9,10,11,12,13,14,15] have been researched. Most non-wearable gaze tracking systems are designed for computer users in a desktop environment using a near-infrared (NIR) camera and an NIR illuminator.

## 2. Related Works

When a gaze tracking system is working properly in a desktop environment, focused images of the user’s eyes should be captured by the gaze tracking camera. Therefore, it is critical to determine the appropriate viewing angle and depth-of-field (DOF) of the gaze tracking camera. Here, DOF indicates the range of *z*-distances between the user’s eyes and the camera where the camera can acquire a focused eye image.

The viewing angle of a gaze tracking camera should not be very small or very large, as this reduces the system’s performance. If the gaze tracking system adopts a camera lens with a small viewing angle, the eye size in the image may be large, which guarantees accurate detection of the pupil and corneal specular reflection (SR). However, the user’s head movement becomes limited, which can degrade the convenience of the system for the user. Conversely, a camera lens with a large viewing angle allows for large head movements by the user, enhancing the convenience of using the system. However, in this case, the size of the eye region in the captured image decreases, which may degrade the detection accuracy of the pupil and corneal SR, and consequently, the gaze detection accuracy.

In addition, when using a lens with a small DOF, the eye region in the captured image is easily blurred by movement of the user’s head in the *z*-direction (approaching or receding from the camera). Blurred eye images can reduce pupil and corneal SR detection accuracies, and thus, the overall gaze detection accuracy. In order to increase the DOF, a lens with a large *f*-number should be used. In general, *f*-number is inversely proportional to the iris size of the lens, or the lens diameter [21]. Therefore, a lens with a large *f*-number has a small diameter, which decreases the brightness of captured images, and the eye region in the image can become too dark to be useful for gaze detection. Therefore, determining the optimal viewing angle and DOF of lenses designed for gaze tracking cameras is a difficult procedure and a prerequisite for realizing high-performance gaze tracking systems.

Nevertheless, most research has focused on algorithm design for improving the accuracy of gaze tracking systems in situations with limited head movement; therefore, previous work implemented gaze tracking cameras without ground truth information for the optimal viewing angle and DOF of the camera lens [1,2,3,4,5,6,7,8,9,10,11,12,13,14,15,16,17,18,19,20]. Previous research [22] has proposed a long-range gaze tracking system that allows for large head movements. However, this work requires gaze tracking cameras with complicated panning and tilting mechanisms in addition to two wide-view cameras. In addition, this gaze tracking camera is also designed without ground truth information for optimal viewing angle and DOF of the camera lens. There have been commercial gaze trackers [23,24]. However, they did not present ground truth information (the experimental measurements of the amount and velocity of user’s head movements) in public, either, although this information is necessary for designing an optimal gaze tracking camera. Therefore, we address this problem providing an empirical study to design an optimal gaze tracking camera based on experimental measurements of the amount and velocity of user’s head movements. Compared to previous works, our research is novel in three ways.

1. To our best knowledge, most previous researches implemented gaze tracking cameras without a ground truth information for determining the optimal viewing angle and DOF of the camera lens [1,2,3,4,5,6,7,8,9,10,11,12,13,14,15,16,17,18,19,20,22]. Eye-tracker manufacturers might also use ground truth information, but they do not provide this in public [23,24]. Therefore, we address this problem providing the empirical research for designing an optimal gaze tracking camera based on experimental measurement of the amount and velocity of user’s head movements.
2. Accurate measurements of the amount and velocity of user’s head movements are made using a web-camera and an ultrasonic sensor while each user plays a game, surfs the web, types, and watches a movie in a desktop environment.
3. Based on the amount and velocity of user head movements, we determine the optimal viewing angle and DOF of the camera lens. We design a gaze tracking system with such a lens and the accuracy and user convenience of our gaze tracking method is compared with those of previous methods.

Recently, various depth-cameras have been commercialized, and Kinect (versions 1 and 2) sensors (named as “Kinect 1.0” and “Kinect 2.0”) have been widely used due to their superiorities of performance. However, the minimum *z*-distance that can be measured by Kinect 1.0 is about 70 cm between object and Kinect 1.0 [25]. The *z*-distance ranges from 40 to 100 cm when people are usually doing various tasks in desktop computer environments. Therefore, accurate *z*-distance in the range from 40 to 70 cm cannot be measured by Kinect 1.0 in our experiments. In addition, because Kinect 1.0 provides the depth map of *z*-distance including the areas of head, neck and body, the face region which is used for *z*-distance measurement should be separated from these areas through further processing using the visible light camera image of Kinect 1.0. In addition, Kinect 1.0 measures the *z*-distance based on trigonometry by using the patterned light produced by laser projector which has the feature of straight [25]. Therefore, in the case that the depth map of face is obtained at near *z*-distance (from 40 to 70 cm), the holes in the depth map occur due to the occlusion of light by the protruded area of face such as nose. Thus, the further processing of hole filling should be performed with the depth map of face for *z*-distance measurement [26,27,28]. To overcome these problems, Kinect 2.0 is recently commercialized and it can measure the *z*-distance by using the time-of-flight (TOF) camera. However, Kinect 2.0 shows the low accuracy (with the large variation) of *z*-distance measurement at the near *z*-distance range of 50–100 cm, and authors also told that less reliable *z*-distance is measured by Kinect 2.0 at this near *z*-distance range [29]. Therefore, it cannot be used for *z*-distance measurement in our experiment. 

Kinect devices can be used behind the monitor screen. Considering the *z*-distance range of 40–100 cm in our experiments, Kinect 1.0 should be placed at the position of 30 cm behind the monitor screen. In the case of Kinect 2.0, it should be placed at the position of at least 60 cm behind the monitor. In this case, Kinect device should be placed behind at the upper, left or right position of monitor because monitor should not hide the depth camera of Kinect device. Therefore, additional complicated calibration (among monitor, our web-camera, and the depth camera of Kinect device) should be performed due to the disparities of coordinates of monitor, web-camera, and depth camera. We can consider the method of using the RGB camera of Kinect device. However, the measurement resolution of head movement by the RGB camera becomes too small due to the far *z*-distance between camera and user, and the distorted image of user’s face can be captured. This is because the Kinect device is placed behind at the upper, left or right position of monitor. 

**Figure 1:**
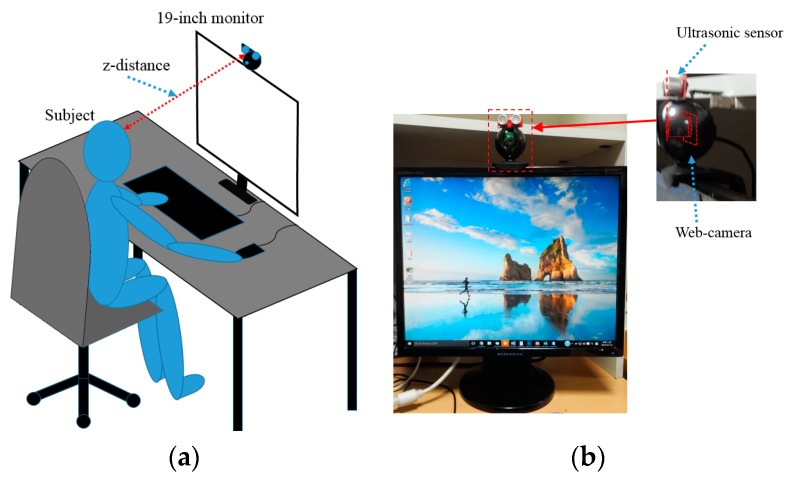
Experimental environment of our method: (**a**) conceptual diagram; and (**b**) example of setup of web-camera and ultrasonic sensor on the 19-inch monitor.

However, in our system, the position of ultrasonic sensor is close to web-camera, and they are placed in the same plane to monitor screen as shown in Figure 1. Therefore, additional complicated calibration is not necessary in our experiments. In the case of SoftKinetic device [30], its speed for measuring depth data becomes slower (lower than 15 frames/s) according to the increase of *z*-distance, which cannot measure the natural head movement of users (our system can measure it at the speed of 30 frames/s).

In addition, the prices of these systems are expensive, i.e., more than $300 in the case of Intel RealSense F200 [31], more than $150 in the case of SoftKinetic device, more than $270 in the case of Kinect 2.0, and about $150–$200 in the case of Kinect 1.0. However, our system (including web-camera and ultrasonic sensor) costs less than $50, and researchers can easily use our system for obtaining the ground-truth information for implementing optimal gaze tracking camera.

Polhemus position tracking sensor (Patriot sensor [32]) can be considered for measuring the *z*-distance of user’s face. However, the sensor should be attached on user’s face during the experiments (performing four tasks (playing a game, surfing the web, typing, and watching a movie), respectively, for about 15 min), which gives much inconvenience to the participants of experiments, and prevents the natural head movement of each user. 

Table 1 compares the previous and proposed methods.

The remaining content of this paper is organized as follows: In Section 3, the proposed system and method for measuring the amount and velocity of a user’s head movement are described. In Section 4, experimental results are presented and the performance of our gaze tracking method is evaluated. Finally, the conclusions of this paper are presented in Section 5.

## 3. Proposed System and Method for Determining the Optimal Viewing Angle and DOF of a Camera Lens

### 3.1. Experimental Environment and Overall Procedures of the Proposed Method

This section gives an overview of our process for detecting a user’s head movement information. The experimental environment of our method is shown in Figure 1. The web-camera and the ultrasonic sensor were placed in the same plane as shown in the right image of Figure 1b.

**Figure 2:**
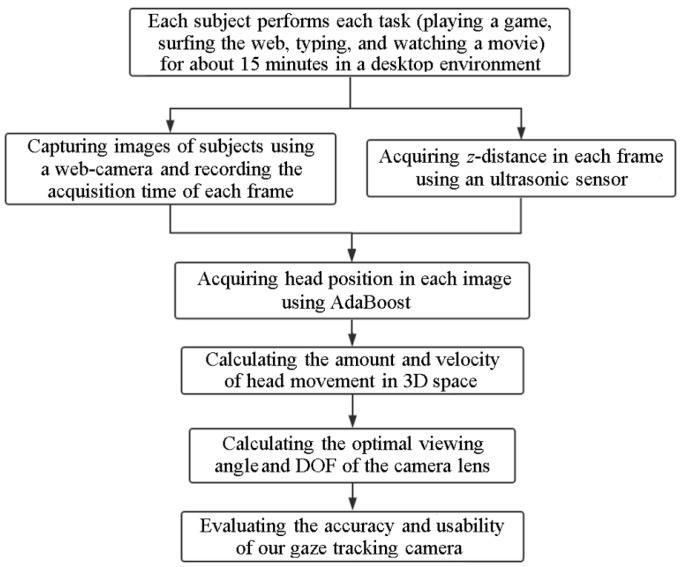
Overall procedure of the proposed method.

As shown in Figure 1 and Figure 2, each participant in our experiments performs four assigned tasks that are common for desktop computer users: playing a game, surfing the web, typing, and watching a movie. While each participant performs each task for about 15 min (for a total of about 60 min of participation per user), images of the participant and the *z*-distance (the distance between the user and the gaze tracking system) are acquired by web-camera and ultrasonic sensor, respectively. User’s head position in an image is located using adaptive boosting (AdaBoost) method [33,34]. The *x*- and *y*-axis coordinates of the user’s head in 3D space are measured based on a pinhole camera model [35] and the *z*-distance measured by the ultrasonic sensor [36]. With this information, the amount of user’s head movements in 3D space can be measured while the user is performing the four tasks. To measure the velocity of user’s head movements, the acquisition time of each image frame is recorded. The velocity of the user’s head movement in 3D space can be calculated with the user’s head position and the acquisition time of each image frame.

With measurements of the amount and velocity of user’s head movements in 3D space, the minimum viewing angle of the camera lens needed for our gaze tracking system can be calculated based on the maximum amount of user’s head movements in the *x*- and *y*-axes. In addition, the optimal DOF of the camera lens for our gaze tracking camera can be determined based on the measured *z*-distances. With this information, we design a lens for our gaze tracking camera. In addition, we evaluate the accuracy, user convenience and interest of our gaze tracking system compared to those using other camera lenses and a commercial gaze tracking system.

### 3.2. Acquisition of z-Distance of the User’s Head by Ultrasonic Sensor

The *z*-distance, the distance between the gaze tracking system and the user’s face, is measured using an SRF04 ultrasonic sensor [36] installed under the web-camera as indicated in Figure 1. The sensor is composed of two parts—the receiver and transmitter—and a control board. The receiver and transmitter are connected to the control board via an I2C interface and the control board is connected to a desktop computer via a universal serial bus (USB) 2.0 interface. Therefore, the *z*-distance is measured by the receiver and is seamlessly transmitted to the desktop computer by the control board at 40 kHz, a frequency at which the *z*-distance can be measured even in the case of rapid head movements.

**Figure 3:**
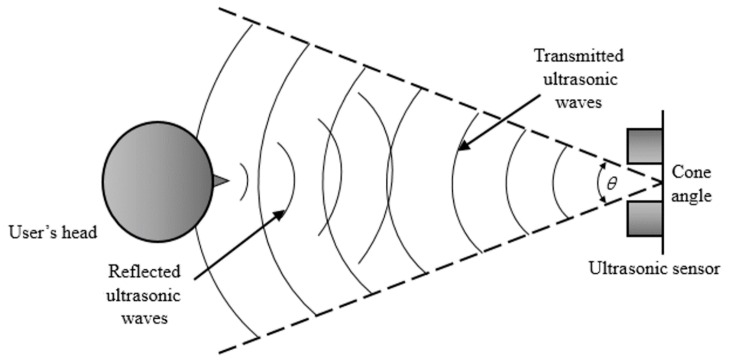
The measurement of *z*-distance with an ultrasonic sensor.

The measurable range of the ultrasonic sensor is from 3 cm to 3 m. The cone angle (*θ* in Figure 3) of the ultrasonic beam is from about 80° to 140°. As illustrated in Figure 3, the *z*-distance of a user’s head is measured using a transmitted pulse of sound whose frequency (40 KHz) is outside of the audible range (about 20 Hz–20 KHz [37]) of humans. The pulse of ultrasonic sound is generated by the transmitter and reflected back from the surface of user’s face in the path of the ultrasonic wave to the transducer. The time between transmission and receipt of the wave can be measured and used to calculate the *z*-distance. 

Before the experiments for measuring *z*-distance of user’s face, we measured the accuracy of ultrasonic sensor with calibration board and actual faces of five people, respectively. When using the calibration board (perpendicular to the ultrasonic sensor) according to the *z*-distance from 40 to 100 cm (from the ultrasonic sensor), the error between ground-truth *z*-distance and measured one by the sensor was less than 1 cm. Here, the ground-truth *z*-distance was measured by laser-distance measurement device [38]. In addition, with the actual faces of five people (who did not participate in the experiments of Section 4) according to the *z*-distance from 40 to 100 cm, the error between ground-truth and measured *z*-distances was about 2 cm. It means that the *z*-distance measured by the ultrasonic sensor is smaller by 2 cm than the ground-truth *z*-distance in most cases. Therefore, we used the compensated *z*-distance (*z_comp_*) based on the Equation (1) in our experiments of Section 4.

(1) $$z_{comp}=z+2$$

If the extended arm includes the beam range of 80° to 140°, incorrect *z*-distance can be measured. In order to solve this problem, we set the beam range so as to include only the user’s head (not including other parts of body) by manually adjusting the direction of ultrasonic sensor according to the sitting heights of user. During this adjustment, we told the participant to keep their head still, even though it takes little time (less than 10 s). From that, the other parts of body (even with the extended arms forward) were not included in the beam range. This step of adjustment was performed just one time (per each participant) before the experiments, and each participant can freely move his or her head after this step. By conclusion, during the experiments, we did not tell the participants to keep their head still, and they can freely move his or her head.

Nevertheless, in the case that the user raises hand close to his face, incorrect *z*-distance from the hand can be measured, which causes the incorrect measurement of user’s head position and movement. To solve this problem, we manually deleted the data in this case by observing the captured video of user in our experiment.

In addition, we compensated the measured *z*-distance by the height of nose (by using the manually measured height of nose as the offset value for compensation) for more accurate measurement of *z*-distance of user’s face.

### 3.3. Acquisition of User Face Images and Measurement of the Amount of Head Movement

**Figure 4:**
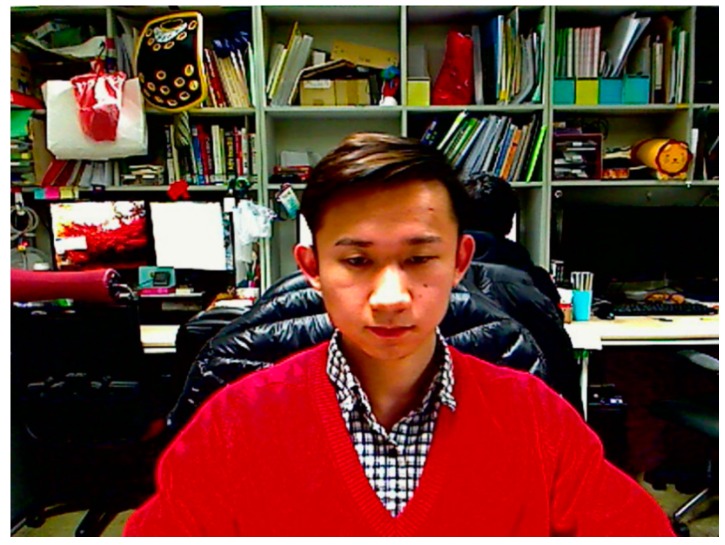
Example of a captured image.

An unconstrained experimental environment is required for measuring the natural head movements of users [33]. Thus, images of user’s faces were captured at 30 frames/s using a web-camera (Logitech C600 [39]) installed at the top-center position of the monitor as indicated in Figure 1. An example of a captured image is shown in Figure 4. The size of the image is 1600 × 1200 pixels and the image is captured with RGB colors in three channels. 

It is not simple to directly measure the user’s head position in 3D space; therefore as a first step, we detect the user’s face position using AdaBoost, implemented using the OpenCV library (version 2.4.2, Intel Corporation, Santa Clara, CA, USA [40]), in the acquired images. Faces are detected using the provided Haar classifier cascade with a scale factor of 1.2. The face detection results by AdaBoost method were manually checked again, and incorrectly detected results were excluded for measuring accurate head movement in our experiments. From that, we can enhance the reliability of measured head movement in our experiments. Then, we calculate head position in 3D space based on a pinhole camera model and the *z*-distance measured by the ultrasonic sensor.

**Figure 5:**
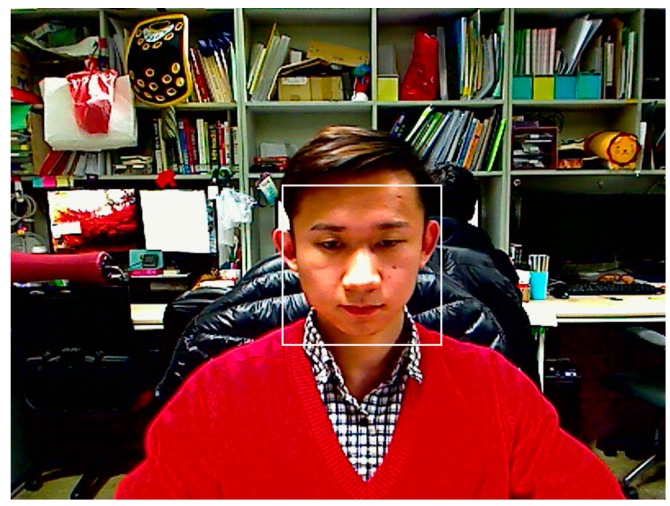
Example of the detected face region (*z*-distance: 61 cm, center coordinate of face region: (859, 627)).

An example of the detected face region is shown in Figure 5. The center position of the face region is used as the user’s head position in image. With the head position information in continuous image frames, we can measure the amount of head movement in images.

**Figure 6:**
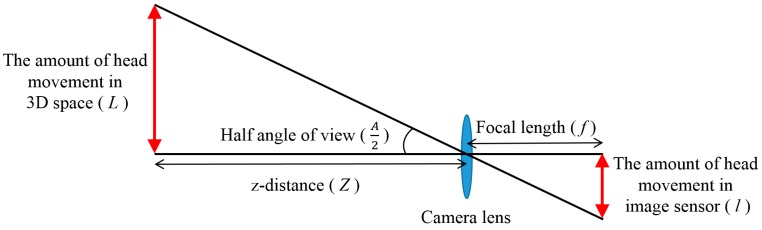
Measuring the amount of head movement in 3D space based on pinhole camera model and *z*-distance.

The amount of user’s head movements in 3D space can then be calculated based on a pinhole camera model and the *z*-distance measured by the ultrasonic sensor. The pinhole camera model is as follows. As illustrated in Figure 6, the amount of head movement in image sensor (*l*) is measured by the movement of face box detected by AdaBoost method in input images.

With the focal length of the camera lens *f*, which is obtained by camera calibration, we can obtain the amount of head movement in 3D space (*L* of Figure 6) based on Equations (2) and (3). The *z*-distance of the user’s face is measured using the ultrasonic sensor as explained in Section 3.2.

(2) $$\frac{L}{z}=\frac{l}{f}$$

(3) $$L=\frac{l\times z}{f}$$

The focal length of the camera lens *f* is measured as 1362 pixels and the amount of head movement in the image sensor (*l*) is measured from the center point of the face region in pixels. In addition, the *z*-distances measured by the ultrasonic sensor are in centimeters. Thus, the amount of user’s head movements in 3D space is also measured in centimeters based on Equation (3). From this and the maximum amount of user’s head movements in 3D space measured while performing four tasks (playing a game, surfing the web, typing, and watching a movie), the range of camera viewing angles (*A*) can be calculated using Equation (4).

(4) $$A=2{tan}^{-1}\frac{\left|L\right|}{z}$$

### 3.4. Measurement of Head Movement Velocity

After measuring the amount of head movement in images and 3D space, as explained in previous section, we can also obtain the velocity of user’s head movement in images and 3D space, respectively. As depicted in Equation (5), user’s head movement velocity in images ($v_{image}$) is calculated based on the amount of head movement in images (*l*) and the time (*t*) of each image acquisition.

(5) $$v_{image}=\frac{l}{t}$$

(6) $$v_{3Dspace}=\frac{L}{t}$$

In addition, user’s head movement velocity in 3D space ($v_{3Dspace}$) can be calculated using the amount of head movement in 3D space (*L*) and the time (*t*) of each image acquisition, as presented in Equation (6).

(7) $$f\left(x\right)=\frac{K}{\sigma \sqrt{2\pi }}e^{-{\frac{\left(x-\mu \right)}{2\sigma^{2}}}^{2}}$$

In order to remove outliers from the measured velocity data, we use a Gaussian function as presented in Equation (7), which is used for graph fitting of the measured data. In Equation (7), *μ* and *σ* are the mean and standard deviation of the distribution, respectively. *K* is the amplitude factor. 

## 4. Experimental Results

### 4.1. Experimental Environment and Description of Database

Ten participants performed four tasks (playing a game, surfing the web, typing, and watching a movie [41]) for about 15 min in a desktop environment. During experiments, successive images were captured at 30 frames/s using a web-camera (Logitech C600 [39]); the total number of images captured is 110,129. Detailed descriptions of the collected images are shown in Table 2. Experiments were carried out with a desktop computer including an Intel Core2 Quad Q6600 CPU of 2.4 GHz and 4 GB RAM with a 19-inch monitor. The program was implemented in C++ with OpenCV library (version 2.4.2). 

### 4.2. Analysis of the z-Distance of User’s Head

**Figure 7:**
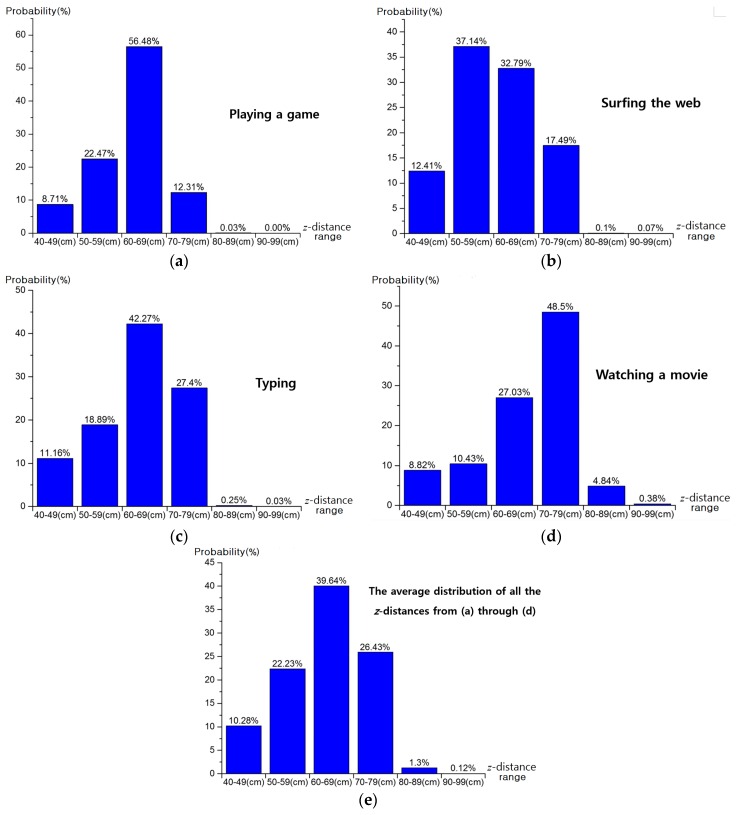
*z*-distance distributions of subjects measured by ultrasonic sensor when subjects were: (**a**) playing a game; (**b**) surfing the web; (**c**) typing; and (**d**) watching a movie. (**e**) The average distribution of all the *z*-distances from (**a**) to (**d**).

With the images collected, the amount of head movement in the z direction is analyzed. As explained in Section 3.2, we acquired *z*-distance data from an ultrasonic sensor for each image acquisition; a total of 110,129 *z*-distance data points were obtained. Most of the *z*-distances range from 40 to 80 cm, as shown in Figure 7 with a range interval of 10 cm. The probability of *z*-distance of Figure 7 is that the frequency of *z*-distance is represented as probability. For example, assuming that all the measured frequencies (*z*-distance) are 2 (40–49 cm), 5 (50–59 cm), and 3 (60–69 cm), respectively. Then, the probabilities (*z*-distance) are 20% (=2/10) (40–49 cm), 50% (=5/10) (50–59 cm), and 30% (=3/10) (60–69 cm), respectively. These probabilities according to *z*-distances are shown in Figure 7.

As shown in Figure 7a, when subjects were playing a game, the most frequent *z*-distances were in the range of 60 cm to 69 cm (56.48%), and almost 100% of *z*-distances were in the range of 40 cm to 79 cm. As presented in Figure 7b, when subjects were surfing the web, the two ranges of 50 cm to 59 cm and 60 cm to 69 cm contained a total of 69.93% of *z*-distance distribution. In addition, 99.83% of *z*-distances were in the range of 40 cm to 79 cm.

When subjects were typing, as shown in Figure 7c, the most frequent *z*-distances were in the range of 60 cm to 69 cm (42.27%), and 99.72% of *z*-distances were in the range of 40 cm to 79 cm. As presented in Figure 7d, when subjects were watching a movie, the most frequent *z*-distances were 70 cm to 79 cm (48.5%). 94.78% of *z*-distances range from 40 cm to 79 cm. 

We did not set the initial *z*-distance of the participants’ head by force. Instead, we requested them to sit in front of monitor naturally at the *z*-distance where they want to be. The reason why the measured *z*-distances of Figure 7 are different for each task is due to the characteristics of task. For example, by comparing Figure 7a through Figure 7d, the *z*-distances when subjects were watching a movie were the largest of all cases. This is because the subjects tended to watch the movie from a greater distance. Figure 7e shows the average distribution of all the *z*-distances from Figure 7a through Figure 7d. The most frequent *z*-distances range from 60 cm to 69 cm (39.64%) and the range from 40 cm to 79 cm included 98.58% of *z*-distances.

Therefore, if the DOF of the camera for gaze tracking could support the range from 40 cm to 79 cm, about 98.58% of user’s *z*-distances can be covered. However, in order to make the DOF of the camera lens cover this wide range of 40 cm (40 cm to 79 cm), the lens diameter will be greatly reduced, which causes the image to be darker [21], and will make pupil detection more difficult. Therefore, in our research, the lens DOF for gaze tracking camera was designed to work in the range from 50 cm to 80 cm, based on the results shown in Figure 7e, about 88.3% of user’s *z*-distances can be covered by our gaze tracking camera.

### 4.3. Analysis of the Amount of User’s Head Movement on the x- and y-Axes

**Figure 8:**
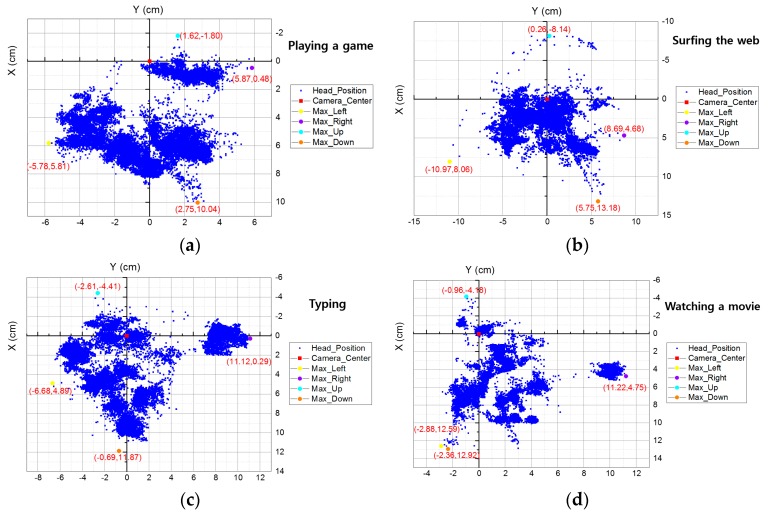

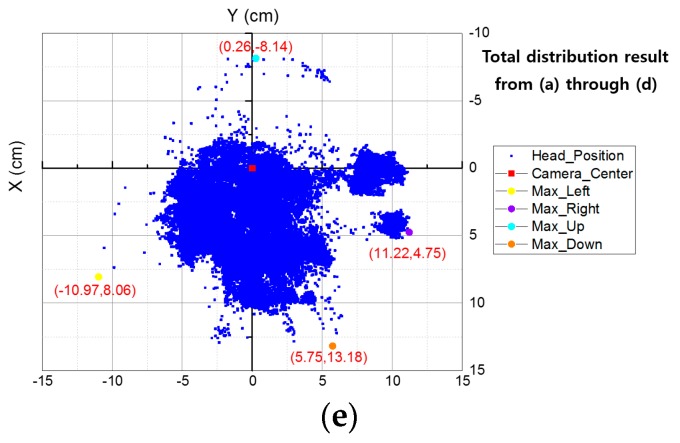
Subjects’ head movements on the *x*- and *y*-axes in 3D space when: (**a**) playing a game; (**b**) surfing the web; (**c**) typing; and (**d**) watching a movie. (**e**) Total distribution result that contains data from (**a**) to (**d**).

The distributions of subjects’ head movements on the *x*- (horizontal direction) and *y*- (vertical direction) axes are presented in Figure 8a through Figure 8d. User’s head movements were acquired while users were performing four tasks, as explained in Section 3.3. The origin of Figure 8 means the *x*- and *y*-position (of 3D space) where the camera axis (passing through the lens center and image (sensor) center of web-camera of Figure 1b) is passing through.

When users were playing a game, the maximum amount of head movement on the *x-* and *y*-axes was 11.65 cm and 11.84 cm, respectively, as shown in Figure 8a. When users were surfing the web, the maximum amount of head movement on the *x*- and *y*-axes was 19.66 cm and 21.32 cm, respectively, as shown in Figure 8b. When users were typing, the maximum amount of head movement on the *x*- and *y*-axes was 17.8 cm and 16.28 cm, respectively, as shown in Figure 8c. When users were watching a movie, the maximum amount of head movement on the *x*- and *y*-axes was 14.1 cm and 17.1 cm, respectively, as shown in Figure 8d.

The result of total distribution from Figure 8a to Figure 8d is presented in Figure 8e; the maximum amount of head movement on the *x*- and *y*-axes was 22.19 cm and 21.32 cm, respectively. As shown in Figure 6, the angle of camera view can be calculated using the amount of head movement (*L*) and *z*-distance (*z*) with Equation (4), presented in Section 3.3. For example, the angle of camera view (*A*) is calculated as 90° in the case that *L* and *z* are same as 60 cm based on the Equation (4).

Based on that equation, the calculated maximum angle of camera view required for four cases were described in Table 3, and the maximum angle of camera view for all the cases was determined as 20.72° (when subjects were web surfing). Therefore, the lens of our gaze tracking camera was designed so as to cover the viewing angle of 20.72°. In addition, all the measured results of Figure 7 and Figure 8 are summarized in Table 3.

We then measure the angle of view of three other cameras in addition to the web-camera (Logitech C600 [39]) with a variety of camera lenses. We used three high-speed cameras (Point Grey [42]): the Gazelle, Grasshopper3, and Flea3 models.

Lenses of focal lengths 9 mm through 50 mm were applied to the four cameras. The angles of camera view for each camera and lens are shown in Table 4. In general, with lenses with a smaller focal length, the consequent angle of camera view increases, as shown in Table 4.

Based on the above results of Figure 8 and Table 3, the angle of camera view should be at least 20.72°. However, if the angle of the camera view is much larger than 20.72°, the camera captures larger area in the image, which means the size of object becomes smaller in the captured image. Consequently, the size of user’s eye is also smaller and the image resolution of user’s eyes in the captured image decreases. With the smaller eye image, the image sizes of pupil and corneal SR also become smaller, and the accuracy of detecting the pupil and corneal SR is inevitably decreased. Because the gaze position is calculated based on the relative position of pupil center and the center of corneal SR (see details in Section 4.5), the final gaze detection accuracy is also decreased. Considering these, we prefer an angle of view similar to 20.72° for our gaze tracking camera. Based on this criterion, we can confirm that the 25-mm lens applied to the Gazelle and Grasshopper3 cameras has a sufficient angle of view for our gaze tracking system. In the case of the Flea3 camera and the C600 web-camera, the 9-mm lens is sufficient to cover user’s head movement.

### 4.4. Analysis of User’s Head Movement Velocity

**Figure 9:**
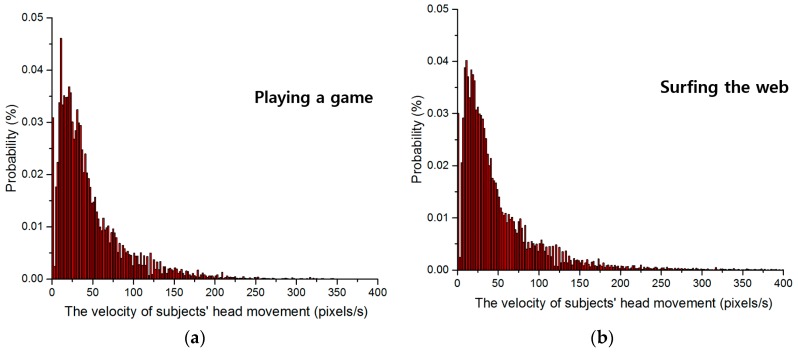

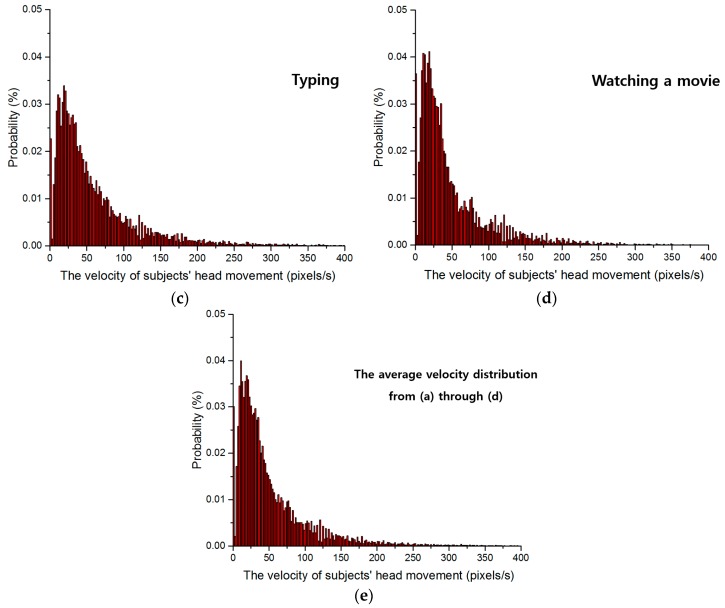
Subjects’ head movement velocities in successive captured images while: (**a**) playing a game; (**b**) surfing the web; (**c**) typing; and (**d**) watching a movie; (**e**) The average velocity distribution from (**a**) to (**d**).

**Figure 10:**
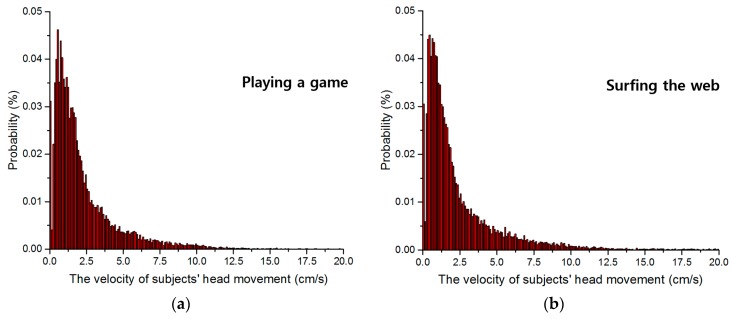

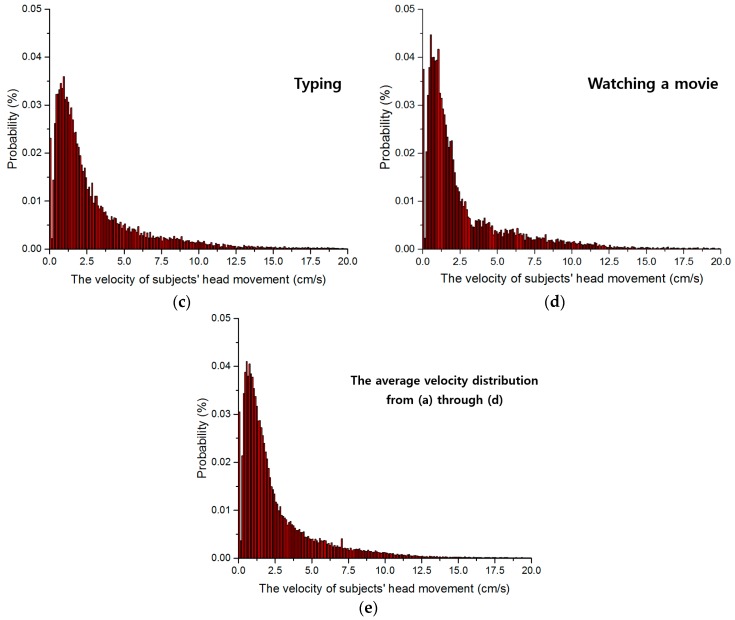
Subjects’ head movement velocities in 3D space while: (**a**) playing a game; (**b**) surfing the web; (**c**) typing; and (**d**) watching a movie; (**e**) The average velocity distribution from (**a**) to (**d**).

Subjects’ head movement velocity was calculated using Equation (5). Velocities in successive captured images and in 3D space are shown in Figure 9 and Figure 10, respectively. The probabilities of velocities in the images are shown in Figure 9a through Figure 9d for subjects playing a game, surfing the web, typing, and watching a movie, respectively. The average distribution contained in Figure 9a through Figure 9d is also shown in Figure 9e. Figure 10 shows the amount (magnitude) of velocity of user’s head movement in 3D space (not 3D plot), whereas Figure 9 shows that in 2D image. Therefore, the unit of *x*-axis of Figure 10 is cm/s whereas that of Figure 9 is pixel/s.

Based on the pinhole camera model in Section 3.3, the velocity of subjects’ head movement in 3D space can be calculated. The probabilities of velocities in 3D space are shown in Figure 10a through Figure 10d as subjects performed four tasks. The average distribution contained in Figure 10a through Figure 10d is also shown in Figure 10e. 

As shown in Figure 8, we obtain the *x*- and *y*-movement (as the unit of cm) of user’s head in 3D space based on the *x*- and *y*-movement in image (measured by AdaBoost method) and *z*-distance (measured by ultrasonic sensor). Because we know the time interval between two successive images in our experiments of Figure 8 as about 0.033 (=1/30) s (from the image capturing speed of 30 frames/s), we can obtain the amount (magnitude) of velocity (as the unit of cm/s) of user’s head movement in 3D space. For example, if the *x*- and *y*-movement of user’s head in 3D space is 0.33 cm, the amount (magnitude) of velocity of user’s head movement in 3D space becomes 10 (=0.33 cm/0.033 s) (cm/s). From this, Figure 10a–e were obtained.

The probability of velocity is that the frequency of velocity is presented as probability. For example, assuming that all the measured frequencies of velocity are 2 (at the velocity of 10 pixels/s), 5 (at the velocity of 20 pixels/s), and 3 (at the velocity of 30 pixels/s), respectively. Then, the probabilities of velocity are 20% (=2/10) (at the velocity of 10 pixels/s), 50% (=5/10) (at the velocity of 20 pixels/s), and 30% (=3/10) (at the velocity of 30 pixels/s), respectively. These probabilities according to velocities are shown in Figure 9 and Figure 10. 

The reason why expressing the frequency of velocity measures as a probability is as follows. In the case of expressing it as a frequency, the total sum of all the frequencies are the total number of images. For example, if expressing it as a frequency, the total sum of all the frequencies of Figure 9a (or Figure 10a) is same to the total number of images (28,579) in the case of playing game of Table 2. As shown in Table 2, there exists a little difference among the total numbers of images in each task (playing game, surfing the web, typing, and watching a movie), and the total sum of all the frequencies can be a little different. Therefore, we express it as a probability in order to make the total sum the same (as 1) in all of the Figure 9a–d (or Figure 10a–d). From that, more accurate analysis of head velocity can be possible without the bias caused by the difference of the total number of images.

For statistical analysis and outlier removal, we fit a Gaussian function, as in Equation (7), to each velocity distribution. Table 5 and Table 6 show the head movement velocities in successive images and 3D space as analyzed with Gaussian fitting. As explained in Section 3.4, based on the 3*σ* principle, values less than *μ* + *σ*, *μ* + 2*σ*, and *μ* + 3*σ* can be regarded as having confidence levels of 68.27%, 95.45%, and 99.73%, respectively [43].

In this research, we measured only the amount (magnitude) of velocity (not the direction of velocity) of head movement (based on the amount of horizontal and vertical head movement of Figure 8) as shown in Figure 9 and Figure 10. That is because only the amount of velocity of head movement is necessary for determining the size of eye searching region in successive images. As shown in Figure 8, the direction of head movement is random (without any directional trend). Therefore, only the amount of velocity of head movement is used for the determination of eye searching region.

Based on the results in Figure 9 and Figure 10 and Table 5 and Table 6, we determined the size of the region to be searched for eye detection in successive images using the average velocity value at the position *μ* + 3*σ*, which we can expect to contain the eye area in almost 99.73% of cases. As explained in Section 3.4, the parameters of Gaussian fitting of the Equation (7) were adaptively determined according to the different figures of Figure 9 and Figure 10. For example, in the case of Figure 10a, *μ*, *σ*, and *K* are 0.55, 1.62, and 0.19, respectively. For example, in the case of Figure 10c, *μ*, *σ*, and *K* are 0.95, 1.58, and 0.15, respectively.

The *z*-directional velocity of head movement was not measured in our research because it has less effect on the size of eye searching region than the velocity of horizontal and vertical head movement. This can be proved as follows. 

**Figure 11:**
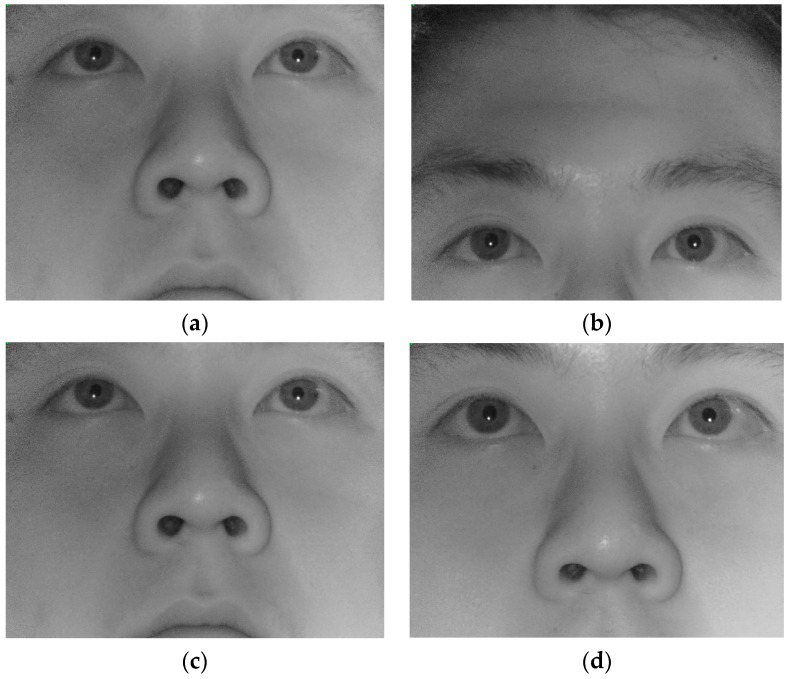
Images captured by our gaze tracking camera: (**a**) before head movement in the vertical direction; (**b**) after movement in the vertical direction; (**c**) before head movement in the *z*-direction; and (**d**) after movement in the *z*-direction (approaching to the camera).

Figure 11a,b, respectively, presents the two images (before and after head movement in the vertical direction) captured by our gaze tracking camera. In addition, Figure 11c,d, respectively, presents the two images (before and after head movement in the *z*-direction (approaching to the camera)) captured by our gaze tracking camera. The movement velocity between Figure 11a,b is the same as that between Figure 11c,d. As shown in these Figure 11, the amount of eye movement in image between Figure 11a,b is much larger than that between Figure 11c,d. This is due to the principle of pinhole camera model ($l=\frac{L}{z}\;f$, based on the Equation (2)). That is, even with the same amount of head movement, the amount of movement in 3D space (*L*) has the larger effect on the measured movement in image sensor (*l*) than that in *z*-direction (*z*) base on this equation. Therefore, the eye searching region should be much larger in the case of horizontal or vertical head movement compared to that of *z*-direction movement.

### 4.5. Performance Evaluation of Our Gaze Tracking System with Analyses

We designed our gaze detection camera and system based on the results in Section 4.2, Section 4.3 and Section 4.4. Since our gaze tracking systems utilized two cameras, the Gazelle and Grasshopper3, with 25- and 35-mm lenses, we compared gaze tracking accuracies for the different hardware. 

In our research, user’s gaze position was calculated based on the detected pupil center and corneal SR center using a geometric transform [44]. Pupil center is located based on image binarization, morphological processing, and ellipse fitting. Corneal SR center is detected based on image binarization, component labeling, and calculation of geometric center of SR area. User’s head movement is compensated based on the relative positional information between the pupil center and corneal SR center. Then, based on the information of user’s calibration (each user gazes at nine calibration points on monitor at initial stage), four matrices of geometric transform are obtained, and user’s final gaze position on the monitor is calculated using these matrices. More detailed explanations of gaze tracking method are included in our previous paper [44]. Because our research is not focused on the gaze tracking method itself, we only refer to [44] for the more detail explanations of gaze tracking system and method.

**Figure 12:**
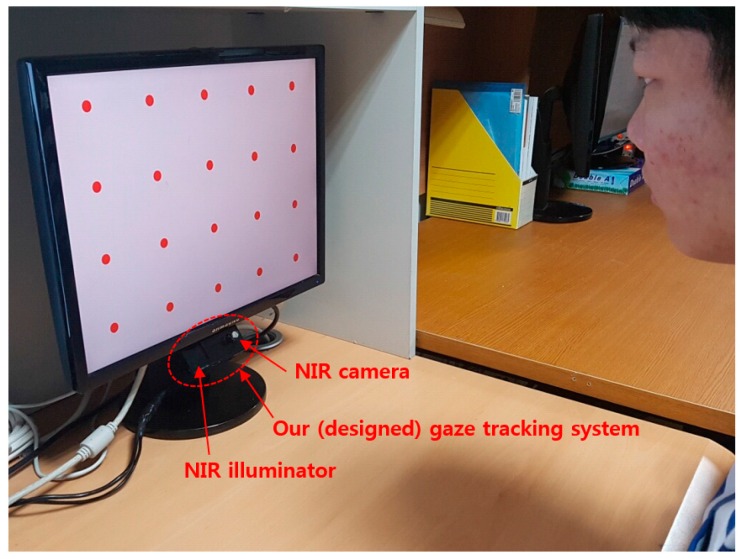
Experimental setup of our gaze tracking system.

The lens DOF for our gaze tracking camera was designed to work in the range from 50 cm to 80 cm as explained in Section 4.2, based on the results shown in Figure 7e. Therefore, the distances between the user’s eyes and the eye tracker were from 50 cm to 80 cm. Those between the user’s eyes and the monitor were also 50 cm to 80 cm because the eye tracker was set below the monitor as shown in Figure 12. As shown in Figure 12, we did not use a chin-rest.

**Figure 13:**
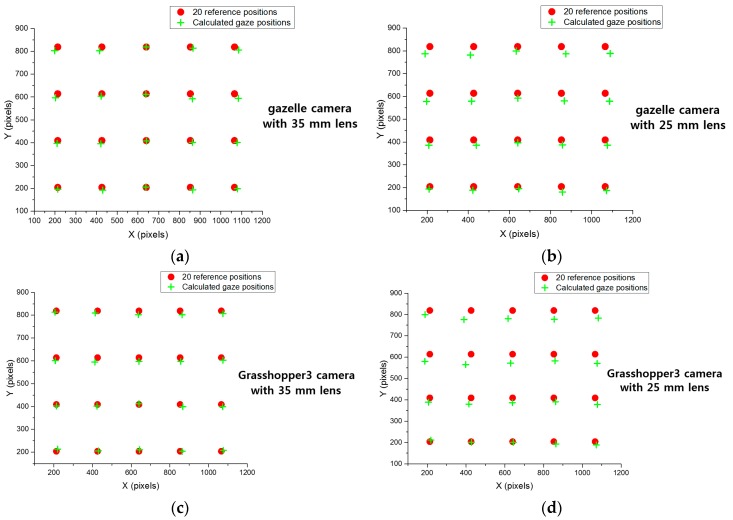
Comparisons of gaze tracking accuracies using: (**a**) gazelle camera with 35 mm lens; (**b**) gazelle camera with 25 mm lens; (**c**) Grasshopper3 camera with 35 mm lens; and (**d**) Grasshopper3 camera with 25 mm lens.

Experiments were performed with images captured when 10 participants looked at 20 reference positions on a 19-inch monitor with a resolution of 1280 × 960 pixels; each participant did five trials. They had no experience with the system of eye-tracking and gaze input. The average gaze tracking accuracy of the 10 subjects (male: 5, female: 5, average age: 26.4, standard deviation of age: 1.71) over five trials each are shown in Table 7. Figure 13 shows gaze tracking accuracies across different cameras and lenses. As shown in Table 7 and Figure 13, we can confirm that gaze tracking accuracies by our system (designed based on the results of Section 4.2, Section 4.3 and Section 4.4) are high, and the average error of gaze tracking is about less than 0.9°. The error is calculated based on the disparity between the reference position and the calculated gaze position.

As shown in Table 4, the 25-mm lens is required to cover the user’s head movements. However, gaze tracking accuracies with this lens were slightly decreased compared to the accuracies when using the 35-mm lens, as shown in Table 7. In detail, in general, with the camera lens of smaller focal length, the angle of camera view increases and larger area can be captured by the camera [45]. Therefore, the larger area can be captured with 25 mm lens compared to the 35 mm lens using same camera, which means the size of object becomes smaller in the captured image with 25 mm lens. Consequently, the size of user’s eye (iris) is also smaller and the image resolution of user’s eyes in the captured image with 25 mm lens decreases compared to the 35 mm lens using same camera as shown in Table 7. With the smaller eye image, the image sizes of pupil and corneal SR also become smaller, and the accuracy of detecting pupil and corneal SR is inevitably decreased. Because the gaze position is calculated based on the relative position of pupil center and the center of corneal SR, the final gaze detection accuracy is also decreased with the lens of smaller focal length compared to that with the larger focal length (if all other conditions are assumed to be the same). To prove this irrespective of the kind of camera, we showed the results with two different cameras as shown in Table 7. Therefore, we posit that lenses whose focal lengths are between 25 and 35 mm are suitable for use in our gaze tracking camera.

In addition, the sizes of user’s irises are also compared in Table 7. Because a camera with a 35-mm lens can have a narrow angle of view compared to one with a 25-mm lens, the size of the iris in an image captured with a 35-mm lens is larger than with a 25-mm lens.

### 4.6. Comparative Subjective Tests on Usability and Analyses

As a final experiment, we compared the usability of our gaze tracking system and a commercial gaze tracking system, TheEyeTribe [23], in terms of user convenience and interest. For experiment, 10 users (who participated in the experiment of Section 4.5) did web surfing for 15 min by our gaze tracking system and TheEyeTribe [23], respectively. For fair comparisons, we requested them to do web surfing using same website (http://www.naver.com) at the same date. Each user moved his gaze position by using our gaze tracking system and TheEyeTribe, respectively, and selected links on the website by clicking mouse button. That is, the participants used a multimodal selection technique by combining gaze position on screen and a manual clicking of mouse left button. Our system and TheEyeTribe provide the feedback about the current gaze position on screen by generating the message of Microsoft Windows operating system (OS) in the case of mouse movement.

**Figure 14:**
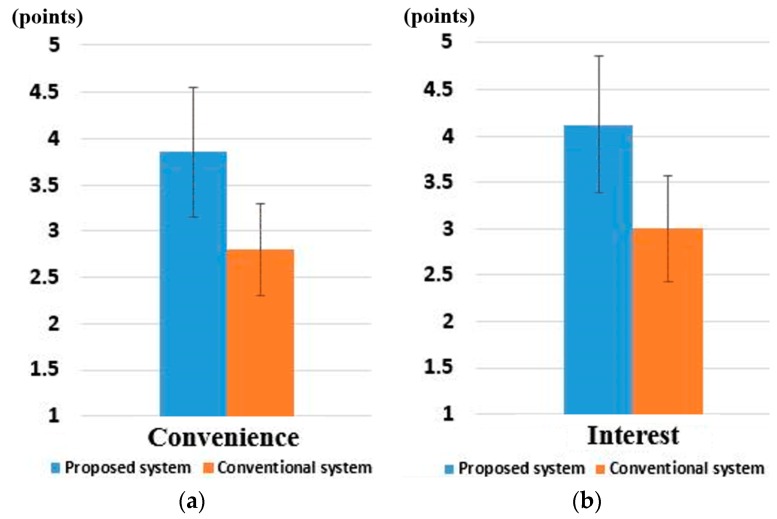
Comparisons of user convenience and interest with our system and conventional system: (**a**) user convenience; and (**b**) user interest.

Then, we asked 10 users (who participated in the experiment of Section 4.5) to rate the convenience of use and their interest in our system and TheEyeTribe on a 5-point scale (5: very high, 4: high, 3: normal, 2: low, 1: very low) by questionnaire. Here, the notion of interest is linked to the amusement the user experience when using one of the systems. Each person tried five times for this procedures (web surfing for about 15 min and subjective test). As explained in Section 4.5, the 10 users were composed of five males and five females with the average age of 26.4 (the standard deviation of age as 1.71). Three users wore glasses, and three users wore contact lenses. The other four users did not wear glasses and contact lens. The *y*-unit of Figure 14 represents these points (from 1 to 5).

As shown in Figure 14a, the users thought that our gaze tracking system is more convenient than TheEyeTribe. It means that people regard that our gaze tracking system is more convenient and easier to be used in the case of moving their gaze position with mouse button clicking in order to do web surfing. In addition, as shown in Figure 14b, the users thought that our gaze tracking system is more interesting than TheEyeTribe. It means that people regard that our system is more interesting in the case of moving their gaze position with mouse button clicking in order to do web surfing.

The lens DOF for our gaze tracking camera was designed to work in the range from 50 to 80 cm, based on the results shown in Figure 7e, about 88.3% of user’s *z*-distances can be covered by our gaze tracking camera. However, according to the specification of TheEyeTribe [23], its operating range is from 45 cm to 75 cm, which is closer to monitor (by 5 cm) compared to our system. As shown in Figure 7e, the probability of user’s *z*-distance is higher than 13% in the range from 75 to 79 cm. Nevertheless, TheEyeTribe cannot cover this *z*-distance range, which lowers the rating scores for convenience and interest. Actually, we asked the participants after the experiments, and they responded that this was the main cause of low rating scores.

In addition, in order to prove that the user convenience with our system is statistically higher than that with commercial system, we performed a *t*-test [46]. The *t*-test has been widely used for statistical analysis for the difference between two measured values. Although the rating based on five-point scale for each system is not very high and database is rather small to perform a significance test, we performed the *t*-test. The *t*-test was performed using two independent samples: user convenience with our system (*µ* = 3.87, *σ* = 0.69) and with the commercial system (*µ* = 2.8, *σ* = 0.5). The calculated *p*-value was about 9.2 × 10^−4^, which is smaller than the 99% (0.01) significance level. 

In general, the null hypothesis for the *t*-test, that there is no difference between the two independent samples, is defined, and the null hypothesis is rejected in the case that the calculated *p*-value is smaller than significance level [46]. The rejection means that there exists significant difference between two independent samples. Therefore, we can conclude that there is a significant difference between user convenience with our system and the commercial system at the significance level of 99%.

In addition, the *t*-test was performed using two independent samples: user interest with our system (*µ* = 4.13, *σ* = 0.72) and with the commercial system (*µ* = 3.0, *σ* = 0.59). The calculated *p*-value was about 1.2 × 10^−3^, which is smaller than the 99% (0.01) significance level. Therefore, we can conclude that there is a significant difference between user interest with our system and the commercial system at the significance level of 99%.

And, we performed Cohen’s *d* analysis, by which the size of the difference between the two groups can be shown using the effect size [47]. Cohen’s *d* analysis has been also widely used for analyzing the difference between two measured values. In general, Cohen’s *d* is classified as small at about 0.2–0.3, as medium at about 0.5, and as large at greater than or equal to 0.8. If the calculated Cohen’s *d* is closer to 0.2–0.3 than 0.5 and 0.8, we can say that the difference between measured values has small effect size. If the calculated Cohen’s *d* is closer to 0.8 than 0.2–0.3 and 0.5, we can say that the difference between measured values has large effect size [47].

The calculated Cohen’s *d* about user convenience was about 1.78 (closer to 0.8 than 0.2–0.3 and 0.5), from which we can conclude that the difference in user convenience between our system and the commercial system is a large effect. The calculated Cohen’s *d* about user interest was about 1.72 (closer to 0.8 than 0.2–0.3 and 0.5), from which we can conclude that the difference in user interest between our system and the commercial system is also a large effect.

From the *t*-test and Cohen’s *d* analysis, we can conclude that there is a significant difference in user convenience and interest between our system and the commercial system.

## 5. Conclusions

In this study, we introduce an empirical design for the optimal gaze tracking camera lens based on experimental measurements of the amount and velocity of user’s head movement. Accurate measurements of the amount and velocity of user’s head movement were made with a web-camera and ultrasonic sensor, while each user played a game, surfed the web, typed, and watched a movie in a desktop environment. User’s head position in successive images is automatically located using AdaBoost. The amount and velocity of head movement in 3D space are obtained using a pinhole camera model and the *z*-distance measured by the ultrasonic sensor. Based on these measurements, we determine the optimal viewing angle and DOF of the camera lens. We design our gaze tracking system using a lens meeting these specifications, and the accuracy of our gaze tracking system is compared with those obtained using various other cameras and lenses. In addition, the user convenience of our system is compared to that of a commercial system. The results of our empirical study would be helpful for developers of gaze tracking cameras and systems.

In future work, we will extend our empirical study to other kinds of gaze tracking systems, such as wearable systems or those that include panning, tilting, and auto focusing functionality. In addition, we would perform the comparative experiments using our ultrasonic sensor with commercial depth camera. In addition, although we tried our best to obtain the generalized conclusion from our experiments, there exist various factors not considered in our experiments. Other factors of user’s preferences and activities can affect the results, and we will also consider these factors in future research.

## Figures and Tables

**Table 1:** Comparison of previous and proposed methods for designing a gaze tracking camera.

Category	Method	Advantages	Disadvantage
Without a ground truth information for determining the viewing angle and DOF of the camera lens [1,2,3,4,5,6,7,8,9,10,11,12,13,14,15,16,17,18,19,20,22]	Wearable gaze tracking system [2,3,4,5], non-wearable gaze tracking system with a single camera [1,6,7,8,9,10,16,18,19,20], and multiple cameras [11,12,13,14,15,17,22]	Implementation time is short because the additional procedures of measuring the amount and velocity of user’s head movements are not necessary	Without determining the optimal viewing angle and DOF of the camera lens through empirical study, gaze tracking accuracy can be reduced or user’s head movement can be limited
With a ground truth information for determining the viewing angle and DOF of the camera lens (Proposed method)	The accurate amount and velocity of user’s head movements are measured with a web-camera and ultrasonic sensor for designing a non-wearable gaze tracking system with a single camera	Gaze tracking accuracy can be enhanced without limiting user’s head movements by determining the optimal viewing angle and DOF of the camera lens	Additional procedures for measuring the amount and velocity of user’s head movements are required

**Table 2:** The numbers of images during experiments.

Tasks	Numbers of Images
Playing game	28,579
Surfing the web	28,742
Typing	26,529
Watching a movie	26,279
Total	110,129

**Table 3:** The summarized results of Figure 7 and Figure 8 (in °).

Tasks	Maximum Angle of View on *x*-Axis	Maximum Angle of View on *y*-Axis
Game	13.73	17.69
Web surfing	20.72	18.84
Typing	19.42	19.09
Movie watching	16.54	20.39

**Table 4:** The angles of camera view for each camera and lens (in °).

Focal Length of Lens	Gazelle	Grasshopper3	Flea3	C600 Web-Camera
50 mm	10.63	10.96	3.89	4.51
35 mm	16.5	16.83	6.01	6.52
25 mm	23.83	24.16	8.58	9.27
22 mm	25.25	25.59	9.44	10.57
17 mm	32.5	32.85	12.24	13.56
9 mm	61.12	61.47	22.91	25.21

**Table 5:** The velocities of head movement in successive images through the analysis based on Gaussian fitting (in pixels/s).

Tasks	*μ*	*μ* + *σ* (68.27%)	*μ* + 2*σ* (95.45%)	*μ* + 3*σ* (99.73%)
Game	11	49.39	87.78	126.18
Web surfing	11	47.57	84.15	120.72
Typing	21	60.13	99.25	138.38
Movie watching	19	43.91	68.82	93.73
Average	11	49.66	88.32	126.98

**Table 6:** Head movement velocities in 3D space based on Gaussian fitting analysis (in cm/s).

Tasks	*μ*	*μ* + *σ* (68.27%)	*μ* + 2*σ* (95.45%)	*μ* + 3*σ* (99.73%)
Game	0.55	2.17	3.78	5.40
Web surfing	0.45	1.94	3.44	4.94
Typing	0.95	2.53	4.11	5.69
Movie watching	0.55	1.97	3.39	4.81
Average	0.55	2.14	3.74	5.33

**Table 7:** Comparisons of gaze tracking accuracies and iris diameter in captured image.

Various Cameras with Lenses	Gaze Detection Accuracy (°)	Iris Diameter (Pixels)
Gazelle with 35 mm lens	0.57	117
Gazelle with 25 mm lens	0.84	82
Grasshopper3 with 35 mm lens	0.56	121
Grasshopper3 with 25 mm lens	0.9	87
